# Strong Public Health Recommendations from Weak Evidence? Lessons Learned in Developing Guidance on the Public Health Management of Meningococcal Disease

**DOI:** 10.1155/2015/569235

**Published:** 2015-11-26

**Authors:** Germaine Hanquet, Pawel Stefanoff, Wiebke Hellenbrand, Sigrid Heuberger, Pierluigi Lopalco, James M. Stuart

**Affiliations:** ^1^Consultant Epidemiologist (Independent), 1081 Brussels, Belgium; ^2^Department of Vaccinology, University of Antwerp, 2000 Antwerp, Belgium; ^3^Division of Epidemiology, Norwegian Institute of Public Health, 0403 Oslo, Norway; ^4^National Institute of Public Health-National Institute of Hygiene, 400-791 Warsaw, Poland; ^5^Department of Infectious Disease Epidemiology, Immunization Unit, Robert Koch Institute, 13086 Berlin, Germany; ^6^Meningococcal Reference Laboratory, Austrian Agency for Food and Health Safety, 8010 Graz, Austria; ^7^Vaccine-Preventable Diseases Programme, European Centre for Disease Prevention and Control, 171 83 Stockholm, Sweden; ^8^School of Social and Community Medicine, University of Bristol, Bristol BS8 2BN, UK; ^9^Faculty of Infectious and Tropical Diseases, London School of Hygiene & Tropical Medicine, London WC1E 7HT, UK

## Abstract

The evidence underpinning public health policy is often of low quality, leading to inconsistencies in recommended interventions. One example is the divergence in national policies across Europe for managing contacts of invasive meningococcal disease. Aiming to develop consistent guidance at the European level, a group of experts reviewed the literature and formulated recommendations. The group defined eight priority research questions, searched the literature, and formulated recommendations using GRADE methodology. Five of the research questions are discussed in this paper. After taking into account quality of evidence, benefit, harm, value, preference, burden on patient of the intervention, and resource implications, we made four strong recommendations and five weak recommendations for intervention. Strong recommendations related not only to one question with very low quality of evidence as well as to two questions with moderate to high quality of evidence. The weak recommendations related to two questions with low and very low quality of evidence but also to one question with moderate quality of evidence. GRADE methodology ensures a transparent process and explicit recognition of additional factors that should be considered when making recommendations for policy. This approach can be usefully applied to many areas of public health policy where evidence quality is often low.

## 1. Introduction

The incidence of invasive meningococcal disease (IMD), caused by* Neisseria meningitidis*, is low in Europe, but case fatality is high (0.8 cases/100,000 inhabitants and 8.7%, resp., in 2011) [1]. Outbreaks of IMD may generate significant anxiety in the population, and even a single case may have important public health implications [2, 3].* N. meningitidis* is transmitted from person to person and the risk of disease is highest in contacts from the same household as a case [4, 5]. In 2007, a European survey showed that recommendations for chemoprophylaxis to eliminate nasopharyngeal carriage in close contacts of IMD cases varied across Europe, in particular regarding the type of antibiotic and the groups that should be targeted [6, 7]. Discrepancies were partly due to differences in policy, medical practices, and health systems but could also be explained by uncertainty surrounding the effectiveness of preventive measures or the differences in methods used to develop recommendations [6].

Divergences in national policies are particularly problematic in cross-border settings, as they lead to differences in disease management among population groups with the same exposure. For instance, passengers sharing an aeroplane flight with a case of IMD might or might not receive chemoprophylaxis depending on their country of residence [8]. The European Centre for Disease Prevention and Control (ECDC) therefore commissioned a group of experts to develop guidance for European countries on the management of contacts of IMD.

It was clear from the outset that high quality evidence in this area would be limited as in many areas of public health [9]. This is because randomized clinical trials on public health interventions are often difficult to organize (particularly when incidence of the outcome is low) and the use of placebo is no longer considered ethical when the intervention studied is already a recommended standard of care. In addition, evidence may be indirect as when only surrogate (proxy) endpoints are available. Therefore, information often comes from observational studies that are more prone to bias and are considered to provide a lower quality of evidence [10]. However, such studies can still be used in developing recommendations if systematically researched and graded appropriately [11]. In addition, evidence obtained for a public health intervention in one country may not be fully applicable to another setting, as public health interventions are strongly dependent not only on the epidemiological context but also on cultural and economic context of countries in which they are implemented.

Here we share our experience and lessons learned in using different types and quality of evidence to develop guidance on the public health management of IMD for European countries within a short time period using GRADE methodology. The aim of this guidance, available on the ECDC website, was to assist countries across Europe in making decisions about appropriate measures to control and prevent IMD in contacts of cases at national and subnational levels [12].

## 2. Description of the Process

We adapted existing methods for producing evidence-based guidelines to deal with the short time frame and the scarcity of direct evidence (see Section 2.12) [10, 13–17]. The main steps are described below.

### 2.1. Setting Up Expert Groups

We set up a consortium of national experts: four in the area of epidemiology and public health surveillance and one in the area of microbiology of meningococcal disease. The consortium members represented five EU countries, previously involved in the assessment of national practices for IMD management across the European Union [6]. The consortium identified research questions, developed protocols, identified, assessed, and graded evidence, and formulated and graded recommendations. This work was contracted for completion within 6 months, had a budget of 20,000 Euros, and was conducted through two face-to-face meetings, three teleconferences, and close to 500 e-mail exchanges. Each member of the expert group completed a declaration of potential conflict of interests.

The consortium identified other national epidemiologists and microbiologists working with meningococcal disease from all EU countries through two established European networks (the European Meningococcal Disease Society and the ECDC European Invasive Bacterial Diseases Surveillance Network). These EU experts were asked to provide any related grey literature and technical advice during the process. Additionally, the consortium consulted two patient group networks, both based in the United Kingdom, on patient-related values and preferences.

### 2.2. Defining the Area of Guidance and Formulating the Research Questions

The consortium defined research questions for guidance focused on the prevention of subsequent cases following sporadic cases of IMD and based on the needs identified through two previous surveys among public health representatives of EU countries [6, 18]. Five research questions for evidence assessment are discussed in this paper. A summary of the evidence and recommendations for all research questions can be found in the ECDC guidance [12].

*Research Questions for Evidence Assessment*

- What is the effectiveness of chemoprophylaxis to a case of IMD before discharge from hospital in preventing further cases of IMD?
- What is the effectiveness of chemoprophylaxis to household contacts of an IMD case in preventing further cases?
- What is the effectiveness of chemoprophylaxis to contacts of an IMD case in pre-school and school settings in preventing further cases?
- What is the effectiveness of chemoprophylaxis to those sharing the same transport vehicle as an IMD case in preventing further cases?
- Which antibiotic regimes are most effective in eradicating carriage among adults, children and pregnant women?

### 2.3. Defining the Methodology

We opted for GRADE (Grading of Recommendations Assessment, Development, and Evaluation) methodology to assess evidence and produce guidance; we used the GRADE guidance available at the time of this study (2008-09) [14, 17, 19]. GRADE not only considers the balance between the benefits and harm and the quality of evidence but also includes additional factors on which to base recommendations, such as burden of the intervention on the patient, patients' values and preferences, and resource implications, which were not addressed by a number of other grading systems. GRADE also provides clear criteria to qualify the strength of a recommendation. Although GRADE has been considered by some as being too resource intensive and difficult to apply in public health guidance, especially under time constraint and when evidence is limited [20], it is recognized by others to provide a systematic approach, promote dialogue, and ensure documentation of the process that leads to a given recommendation [21, 22]. This makes decision-making more transparent. We referred to previous experience reported in two World Health Organization (WHO) publications on rapid advice guidelines that made use of GRADE methodology and to Cochrane guideline for public health interventions [14, 15, 23].

The consortium developed protocols, templates, and checklists for screening abstracts/papers retrieved in the literature searches to ensure a homogenous process across the research questions and across reviewers. The process was also reviewed against the criteria for guideline development as defined by the AGREE collaboration [16].

### 2.4. Search Strategy and Selection Criteria for Systematic Reviews

When defining the most suitable terms for the population, intervention, comparison, and outcome (PICO) to define our research questions for the search strategy (see examples in Table 1) [15], we took into account prior knowledge and a preassessment of the literature. Our preassessment suggested that measurements of direct outcomes would not be available for several research questions due to the low incidence of IMD. For these questions, we defined and included proxy outcomes in our search strategy (see examples below Section 2.5).

We defined inclusion and exclusion criteria for selecting studies, applied to each research question. All European languages were included to avoid publication biases. As most of our studied interventions either were standard clinical practice or involved rare outcomes, clinical trials had not been conducted for ethical or logistical reasons. We thus did not limit inclusion to experimental studies but also included observational studies that involved comparison groups and case series with at least 10 cases.

We searched MEDLINE, Embase, Global Health, the Cochrane database of systematic reviews, and the Cochrane central register of controlled trials. The search terms for each question were agreed by at least two members of the consortium. Due to the short time frame, we applied some of the strategies previously used in rapid reviews by limiting the search to the period from January 1990 to the date of the literature search (December 2008) and giving priority to systematic reviews [24]. If a relevant systematic review was identified, we only considered abstracts published from the date of search for the last review up to the end of 2008. If no relevant review was identified, we screened all abstracts published from 1990 to 2008. We reviewed full papers of abstracts identified as relevant. One reviewer only was involved in reviewing abstracts and full papers of each research question due to time constraints. In case of doubts, ad hoc opinion of a second reviewer was requested.

We examined reference lists of the selected papers for other relevant publications and searched Google Scholar for citations of identified key papers. For instance, for the research question on effectiveness of antibiotic regimens (Question E), we found a nonindexed but peer reviewed trial on antibiotic prophylaxis by using Google Scholar, though this study had not been retrieved by a previous Cochrane systematic review [25].

### 2.5. Use of Indirect Evidence

As studies measuring direct evidence on outcomes could not be found in four of the five research questions discussed in this paper, we defined and searched for indirect evidence on outcomes. For example, the relevant direct outcome for the question on the effectiveness of chemoprophylaxis before hospital discharge in preventing further cases among contacts (Question A) would be the incidence of subsequent cases in household contacts of the IMD patients who received antibiotics prior to discharge from hospital. A prior systematic review did not identify relevant studies measuring this outcome but showed that eradicating nasopharyngeal carriage in household contacts reduced the risk of further cases [7]. We thus searched for data on the proxy outcome, that is, the prevalence of meningococcal carriage in discharged patients (Table 1).

We also did not find direct evidence on the research question regarding whether chemoprophylaxis of contacts in school settings would prevent further cases (Question C). However, we obtained indirect evidence by comparing the risk of subsequent cases in school contacts (not receiving chemoprophylaxis) with the background incidence rates of IMD in the relevant population [26].

Even when the literature search provided direct evidence on the benefits of an intervention (e.g., effectiveness in preventing secondary cases), the evidence was often insufficient on its harm. In particular, direct evidence on the adverse events of antibiotics administered as chemoprophylaxis (Question E) was insufficient, but we found and reviewed indirect evidence on adverse events of these antibiotics when administered for indications other than chemoprophylaxis (e.g., ciprofloxacin used in cystic fibrosis).

### 2.6. Analysis of Extracted Data

**Figure 1:**
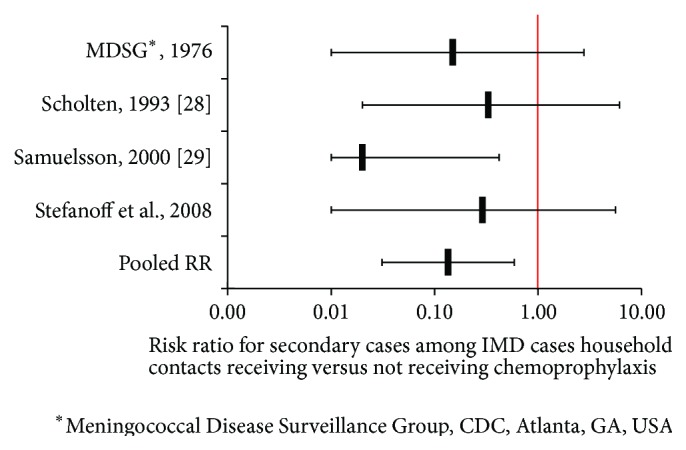
Estimate of effect of chemoprophylaxis to household contacts following a sporadic IMD case.

We extracted and summarized the evidence on benefits and harm and prepared evidence profiles. When possible, we pooled estimates retrieved from selected studies. For instance, for Question B on chemoprophylaxis for household contacts, we extracted results from a recent study published after a systematic review and analysed these together with the three former studies from the review [7, 27–29]. As the results of the four studies were statistically homogeneous, we calculated a common pooled estimate (Figure 1) [12]. In case of heterogeneity between studies, we performed stratified analysis when possible. In particular, analyses on the effectiveness of chemoprophylaxis to contacts of an IMD case in preschool and school settings (Question C) were stratified by each educational setting [26].

If the retrieved systematic reviews did not provide the level of detail needed to calculate pooled estimates of effectiveness or to fully answer the research questions, we extracted the necessary data from primary studies when appropriate. For instance, in the research question on antibiotic regimes for different subgroups (Question E), we identified Cochrane systematic review on antibiotics for preventing meningococcal infections, but this review did not present detailed analyses by antibiotic dosage and duration of therapy and did not stratify estimates by subgroups such as children and pregnant and lactating women [25]. We thus retrieved the nine primary studies that involved different dosages, treatment durations, and subgroups, extracted the required data, and appraised the studies based on the full papers.

### 2.7. Reviewing and Grading Evidence

Evidence was classified for all questions as either direct or indirect. We graded bodies of evidence according to GRADE guidelines and classified them as high, moderate, low, or very low, based on study design and quality, inconsistency, indirectness, imprecision, and strength of the association [17, 19]. In particular, we made a judgment of whether the evidence was sufficiently indirect to warrant downgrading. For example, we downgraded the quality of evidence for the four studies retrieved for the research question on chemoprophylaxis for cases before hospital discharge (Question A), as they measured a proxy outcome, had very small study populations ranging from 14 to 51, and used different therapeutic antibiotic regimes. As observational studies start with “low quality” evidence rating according to GRADE [13], this led to classification of the evidence quality to “very low.”

We found high or moderate quality evidence for only two of the five research questions, that is, regarding chemoprophylaxis to household contacts (Question B) and antibiotic regimes (Question E). Only low or very low quality of evidence was found for the remaining three research questions (Questions A, C, and D). Table 1 describes evidence review and grading for three research questions (Questions A, D, and E) for which the quality of evidence was very low (A and D) or moderate to high (E).

### 2.8. Burden on Patient, Values, Preferences, and Resource Implications

Because these factors varied across settings [21, 22], we first outlined, for each question, what should be considered from the patients perspective as burden of the intervention and their values and preferences and resource implications in an EU setting. For instance, for the burden of prophylactic antibiotics (Question E), we considered for each antibiotic the potential side effects, inconvenience (e.g., number of days of treatment) for contacts, ease of administration, and the number of contacts needed to be treated (where possible to calculate) to prevent one IMD case among contacts according to each setting. We also considered the implications of contact tracing, as this can lead to considerable costs when, for instance, tracing close contacts on the same aeroplane as an IMD case is required (Question D) and may even not be feasible, for example, in case of free seating.

We found little information in the literature on burden of intervention perceived by patients and on their values and preferences. We searched for alternative data sources: for instance, information on perceived burden and values was requested from EU experts and national IMD representatives as well as from two meningitis patient associations. This confirmed that IMD is perceived as a severe disease that generates a high level of anxiety, and thus prevention measures are widely accepted, even if associated with some level of discomfort.

### 2.9. Developing and Grading the Strength of Recommendations

The consortium met face-to-face to develop recommendations according to GRADE, based on the quality of evidence and the balance between the benefits and harm, taking into account burden, values, preferences, and costs (see examples in Table 1).

Recommendations were classified as strong or weak as recommended by GRADE [10, 14, 17]. The GRADE guidance available at the time of developing this guidance did not provide an objective method for assessing the balance between benefit, harm, burden, values, and costs [13, 17]. We decided that the entire consortium should participate and agree on this appraisal process and we included advice of two patients' groups regarding values and preferences related to the recommendations. Based on these criteria, four strong and five weak recommendations for intervention were made for the five research questions. Strong recommendations were made not only in relation to two research questions with moderate to high quality of evidence (Questions B and E) but also in relation to one research question with very low quality of evidence (Question A). The weak recommendations for or against intervention referred not only to two research questions with low or very low quality of evidence (Questions C and D) but also to two aspects of one research question with moderate quality of evidence (Question E).

The strong recommendation for which the quality of evidence on the benefit was very low was related to the research question on chemoprophylaxis of IMD cases before hospital discharge (Question A). Despite the very low quality evidence on the benefits, the consortium considered that harm, cost, burden, and values were strongly in favour of the intervention: the low cost of the intervention, the low number of patients not treated with an eradicating antibiotic regimen prior to discharge, and the potential benefit in reducing risk from a life-threatening disease were balanced against limited harm from antibiotics (Table 1). In general, higher quality evidence is more likely to be associated with strong recommendations than lower quality evidence [11]. However, the GRADE methodology indicates that a particular quality of evidence does not imply a particular strength of recommendation. A number of public health guidances and GRADE clinical guidelines issued strong recommendations in the face of a very low quality of evidence [11, 13, 14, 23, 30]. WHO also considers that strong recommendations can be made despite low or very low quality evidence in specific circumstances, as it is the net result of all relevant factors that are important [21]. For instance, WHO rapid advice guidelines for management of sporadic human infection with avian influenza A (H5N1) virus made a strong recommendation to treat H5N1 patients with oseltamivir, although the quality of the underlying evidence was rated as very low in part because of the severity of the disease [23]. A recent review highlighted that over than half (55%) of strong recommendations in WHO guidelines were based on low or very low confidence in effect estimates [30]. A GRADE guidance published after our review describes five situations in which a strong recommendation is warranted despite low or very low confidence in effect estimates [31]. The most relevant to our review was “when low quality evidence suggests benefit in a life-threatening situation,” as all of our recommendations aimed at preventing a life-threatening invasive infection.

Consensus on the recommendations and grading of their strength was difficult to reach regarding chemoprophylaxis in day care settings (Question C). Here, the quality of evidence was low, and divergent recommendations were in place in the consortium members' native countries. Thus each expert was probably influenced by his/her existing national policy. This highlighted that recommendations are built not only on rigorous scientific reviews but also on expert interpretation and judgment of the evidence. An advantage of the GRADE approach is to promote useful dialogue and ensure transparency by making these value judgments explicit [17, 21].

We involved stakeholders and potential users of the guidance in the final steps. As our aim was to produce guidance that could be adapted to the needs of different EU countries, the draft document was circulated through EU experts and patient groups and reviewed by representatives of EU countries in the ECDC Advisory Forum. The feedback from ECDC and EU experts on the draft report allowed useful additions to the guidance [12]. For instance, we added recommendations on use of antibiotics by lactating women on request from representatives from a patient association.

### 2.10. Implications for Practice

We described how the guidance would potentially change current practice in EU countries. For instance, for the research question (E) on effectiveness of antibiotic regimens in IMD prophylaxis, we described what policy changes would be required and potential obstacles to the implementation of this guidance in a EU setting, based on whether the intervention or the specific drug is available and whether the recommended regimen differs from those currently recommended. In particular, some effective dosages did not correspond to recommendations and formulations available in EU countries and would require a change in current guidance. For instance, high quality evidence was available for the effectiveness of a single dose of 750 mg ciprofloxacin for the eradication of meningococcal carriage. However, in many countries, ciprofloxacin is recommended as a 500 mg single dose, although the effectiveness of this lower dosage has not been assessed in a controlled trial.

### 2.11. Strengths of the Process

The guidance was successfully completed within six months and was approved and endorsed by ECDC in 2010 [12]. The GRADE approach allowed transparent judgments on the quality of evidence and the formulation of recommendations. Our process met most of the criteria for guideline development as defined by the AGREE collaboration (2003 version) [16]. We complied with the following criteria: definition of the scope and purpose, stakeholder involvement, rigor of development, clarity and presentation, application, and editorial independence. On the other hand, our review process did not fulfil criteria pertaining to tools for application and audit.

An advantage of GRADE process in developing public health recommendations is the integrated appraisal of related values, preferences, burden to the patient, and resource implications in addition to quality of evidence and the balance between benefits and harm. Based on GRADE 2004–08 guidance, we made strong recommendations for some areas in which the quality of evidence was low or very low. The long deliberations often required to arrive at final agreement of recommendations were facilitated by frequent communication, mainly by e-mail. It should be noted that GRADE work published later provides a systematic approach by describing circumstances in which a strong recommendation is warranted despite low or very low confidence in effect estimates, but these were not available at the time of developing our guidance [30, 31].

The influence of national policies on the judgment of each consortium expert to formulate recommendations (described above) was dealt with by explicitly discussing each recommendation in the entire group. One advantage of having experts from five EU countries in the consortium was also that they had knowledge of current practices and health systems when considering implications for practice of the guidance.

The development of this guidance led to the identification of areas of uncertainty and research gaps, and we identified priorities for further research in each area. It was also a unique opportunity to progress towards common European health policy. Divergent health policies may cause confusion among the public and the media. The most objective argument for common health policies consists of a systematic and transparent search for and evaluation of available evidence. In 2013, we evaluated the impact of this guidance on the recommendations for public health management of IMD in European countries and found out that 90% of the 31 EU countries or regions found it useful at the national level and that 50% used it to update their national guidelines within the three years following the publication of the guidance [32].

WHO adopted a very similar process for developing evidence-based immunization recommendations, published after we initiated this work [21]. Immunization is an area of public health prevention in which the evidence may also be indirect (e.g., immunological surrogate for clinical efficacy) as in our example. GRADE was selected by WHO because it improves transparency in decision-making, promotes dialogue, and provides opportunities to reassess the evidence as required [21].

### 2.12. Limitations of the Process

The consortium included mostly experts in epidemiology and microbiology. It could have benefited from including clinical experts and members of patient organization groups, but the short time frame was already challenging for finalizing the project.

The limited time (6 months) and available resources imply that our literature reviews could not meet the standards of a full systematic review. In addition, we could not cover all aspects of IMD public health management.

Indeed, the comprehensive application of the GRADE methodology including exhaustive systematic reviews may require substantial resources and more time is often required for rigorous development of guidelines [24]. Thus, in this project, we applied some strategies of rapid reviews, such as focusing on existing systematic reviews, having only one expert for evidence reviews, and limiting the search period [24]. These may have introduced biases in the selection and appraisal of studies [24]. However, we enhanced our searches through inclusion of older studies, searching manually the references of retrieved studies, not restricting literature search by language or database, and asking experts for unpublished data and potentially missed studies. Additionally, though our initial search focused on updating systematic reviews, we nonetheless retrieved relevant primary studies to extract all relevant data, if they were not provided in the systematic reviews. Some authors have suggested that when the timeframe is limited, combination of electronic searching, hand searching of relevant reference lists, and consultation with experts on potentially missed articles may provide the most comprehensive results [24]. In this regard, WHO publication on rapid advice guideline—that met similar time constraints—was a particularly useful reference in helping us to ensure transparency of the process [14]. It is likely that the specialist expertise of those performing the review as well as input from other EU experts minimized the risk that relevant studies would be missed [24].

We relied mostly on systematic reviews (including one Cochrane review) for the quality appraisal of individual studies for Questions B and E [7, 25], but these described the risk of biases and not the other GRADE criteria for assigning grades of evidence [11]. In particular we did not fully appraise bodies of evidence for each outcome for imprecision, also due to limited instructions in the GRADE guidance available in 2008 [17]. New guidance published after our review describes each criterion for appraising evidence more explicitly, including imprecision [33], allowing further downgrading for indirectness, imprecision, and reporting bias [11]. We also did not explicitly define which outcomes were critical to a decision and which ones were important for grading overall quality of evidence [24, 34]. It is likely that the strict application of the newer GRADE guidance could have led to further downgrading of the quality of evidence for some of our outcomes, although this may not have changed our recommendations.

As explained above, one of the challenges was that we only found a low quality of evidence (according to GRADE) in most areas, as evidence from RCTs was only available on the effectiveness of antibiotic regimes in eradicating carriage.

The GRADE guidance required defining the burden of the intervention to the patient as well as patients' values, preferences, and resource implications to aid in the development of recommendations. However, the GRADE guidance available at the time of developing these recommendations did not provide a methodology to collect and appraise the evidence in these areas. We found scarce information in the literature on the burden, values, and preferences surrounding interventions and limited data on cost in a few countries, and these may differ across countries. Although we questioned EU representatives and two UK-based meningococcal patient organizations, a representative survey of patients across Europe would be required for obtaining sound and representative evidence. However, we did not have the resources to initiate a multinational public survey on these issues. Furthermore, the GRADE guidance did not standardize how the data on burden and values should affect the recommendation; this is left to deliberation on the part of the decision-making group and has been criticized as a weakness of the GRADE process [20]. The updated GRADE guidance also provides a more structured way to incorporate values and preferences in the development of recommendations [31].

## 3. Conclusions

We developed evidence-based guidance on the public health management of meningococcal disease for EU countries in a short time frame and with limited resources. A number of recommendations in this guidance were based on a low quality of sometimes indirect evidence due to the impracticability of conducting clinical trials on interventions for outcomes that are rare or that have become standard practice. However, the recommendations were generated systematically and transparently, following GRADE and AGREE standards. This approach, that explicitly integrates additional criteria with the quality of evidence, can be usefully applied to the many areas of public health policy in which quality of evidence is often low or indirect. A recent survey of European countries showed that the majority found the guidance based on this process useful, about half had used the guidance to update their national recommendations, and a higher proportion of countries since 2013 compared to that in 2007 recommended evidence-based measures for IMD public health management [32].

## Figures and Tables

**Table 1:** Summary of findings and recommendations for three research questions.

Assessment of evidence quality						Assessment for recommendations				
Number of studies selected/number of studies reviewed	Design and quality	Inconsistency	Indirectness	Other modifying factors^*∗*^	Grade of evidence	Benefits	Harm	Costs and burdens	Values and preferences	Grade of recommendation
*Research question*: What is the effectiveness of chemoprophylaxis to a case of IMD before discharge from hospital in preventing further cases of IMD?										
*Population*: cases of IMD and their household contacts										
*Intervention*: administration of chemoprophylaxis (including therapeutic treatment if effective in carriage eradication) to case prior to discharge from hospital										
*Comparison*: no chemoprophylaxis administered to case prior to discharge from hospital										
*Outcome*: carriage in IMD cases; incidence of IMD in household contacts										

4/349	All observational. No studies addressed the intervention	Different antibiotic regimens, times of swabbing, sampling, and study populations	Proxy outcome: prevalence of carriage following discharge from hospital	Small sample sizes. Study results are statistically homogenous	Very low	Potential reduction of the disease burden among close contacts of discharged cases	Low risk of treatment side effects	Very low cost and low burden for the patient (oral single dose)	Treatment widely accepted	Strong

*Recommendation*: chemoprophylaxis is recommended for patients with IMD on discharge from hospital unless an antibiotic regimen effective in eradicating carriage was used during hospital treatment										

*Implication for practice*: easy to implement in hospital procedures; treatment is widely accepted										

*Research question*: What is the effectiveness of chemoprophylaxis to contacts of a case of IMD who have shared the same transport vehicle as a case of IMD in preventing further cases among those contacts?										
*Population*: contacts of diagnosed IMD cases sharing the same transport vehicle, for example, plane, boat, bus, and car										
*Intervention*: administration of chemoprophylaxis to contacts sharing transport vehicle, following IMD diagnosis in a case										
*Comparison*: no chemoprophylaxis administered to contacts sharing transport vehicle, following IMD diagnosis in a case										
*Outcome*: incidence rate of IMD in contacts sharing a transport vehicle with IMD cases (up to 30 days)										

7/103	Only reports on sporadic cases and 3 clusters linked to travel. No studies addressed the intervention	No consistency across case reports	Proxy outcome: risk of subsequent cases among fellow passengers whether prophylaxis was given or not	No studies clearly established evidence of transmission in transport vehicles	Very low	No evidence of reduction of subsequent cases among contacts sharing the same transport and taking prophylaxis	Low risk of treatment side effects but potential anxiety among those not receiving prophylaxis if targeted	Low cost of the intervention. However, contact tracing can lead to considerable cost and may not be feasible	Treatment likely to be accepted even if objective risk is low. Possible public pressure to give prophylaxis	Weak

*Recommendation*: sharing the same transport vehicle as a case of IMD is not, in itself, an indication for chemoprophylaxis										

*Implication for practice*: a consistent European policy is highly desirable because high potential for confusion related to divergent cross-border policies. However, achieving consensus may not be easy										

*Research question*: Which antibiotic regimes are most effective in eradicating carriage among adults?										
*Population*: adult carriers of *N. meningitidis*										
*Intervention*: administration of antibiotic (type, dose, duration, and route)										
*Comparison*: no antibiotics, alternative type of antibiotic, alternative dose, alternative duration, or alternative route										
*Outcome*: carriage of *N. meningitidis* at ≥7 days of follow-up. Occurrence of resistant strains of *N. meningitidis* after treatment										

28^*∗∗*^	17 RCTs and 3 observational studies; no serious limitations	High consistency of results across studies	Proxy outcome: eradication of carriage. Only assessed in students/army for azithromycin and cefixime	High associations	High: rifampicin, ciprofloxacin, and ceftriaxone Moderate: azithromycin and cefixime	The 5 antibiotics are highly effective (eradication in 79–100%)	Limited harm of antibiotics and mild side effects. Emergence of resistance with rifampicin and interactions with other drugs	Low cost. Lower burden for ciprofloxacin and azithromycin as single oral dose. Ceftriaxone is intramuscular	High acceptability of intervention. A single oral dose is likely to be preferred	Strong for the 5 antibiotics. Weak for “ciprofloxacin, azithromycin and ceftriaxone are preferred”

*Recommendation*: rifampicin, ciprofloxacin, ceftriaxone, azithromycin, and cefixime can be advised for chemoprophylaxis in adults. Ciprofloxacin, azithromycin, and ceftriaxone are preferred										

*Implication for practice*: using ciprofloxacin, azithromycin, or ceftriaxone would require a change of practice in several countries but has a high feasibility at similar or lower cost. Surveillance of resistance is essential										

^*∗*^Strength of association and imprecision [17].

^*∗∗*^Total reviewed not relevant because we identified a systematic review and selected 16 of the included studies as well as 9 studies through references of selected papers. Of the update search conducted in the period after the systematic review, 3/67 studies were included.

RCT: randomized clinical trial; IMD: invasive meningococcal disease.
